# Satisfaction of patients with health care services in tertiary care facilities of Riyadh, Saudi Arabia: A cross-sectional approach

**DOI:** 10.3389/fpubh.2022.1077147

**Published:** 2023-01-13

**Authors:** Nasser Ali Aljarallah, Mansour Almuqbil, Sultan Alshehri, Amro Mohammed Sawadi Khormi, Reshaidan Mohammed AlReshaidan, Fayez Hadi Alomran, Nasser Fawzan Alomar, Fayez Faleh Alshahrani, Majed Sadun Alshammari, Walaa F. Alsanie, Majid Alhomrani, Abdulhakeem S. Alamri, Syed Mohammed Basheeruddin Asdaq

**Affiliations:** ^1^Department of Computer Science and Information Systems, College of Applied Sciences, AlMaarefa University, Ad Diriyah, Riyadh, Saudi Arabia; ^2^Department of Business Administration, College of Business Administration, Majmaah University, Al Majma'ah, Saudi Arabia; ^3^Department of Clinical Pharmacy, College of Pharmacy, King Saud University, Riyadh, Saudi Arabia; ^4^Department of Pharmaceutics, College of Pharmacy, King Saud University, Riyadh, Saudi Arabia; ^5^Department of Pharmacy Practice, College of Pharmacy, AlMaarefa University, Dariyah, Riyadh, Saudi Arabia; ^6^Equame Scientific and Research Center, Riyadh, Saudi Arabia; ^7^Department of Family Medicine, King Abdulaziz Medical City in Riyadh, Ministry of National Guard, Riyadh, Saudi Arabia; ^8^Department of Pharmacy, King Abdulaziz Medical City, Riyadh, Saudi Arabia; ^9^Department of Clinical Laboratory Sciences, The Faculty of Applied Medical Sciences, Taif University, Taif, Saudi Arabia; ^10^Centre of Biomedical Sciences Research (CBSR), Deanship of Scientific Research, Taif University, Taif, Saudi Arabia

**Keywords:** patients' satisfaction, PSQ-18, health care services, quality of care, tertiary care facilities, clinical consultation, Saudi Vision 2030, accessibility of services

## Abstract

As part of Saudi Vision 2030, the country's healthcare system is undergoing a significant makeover, with accessibility and effectiveness serving as the benchmarks for measuring patient care quality. This study's goal was to ascertain the degree of patient satisfaction with the medical care and services received in Riyadh's tertiary care facilities. The PSQ-18 (Patient Satisfaction Questionnaire-18), a standardized validated questionnaire including areas of “overall satisfaction,” “technical quality,” “interpersonal aspect,” “communication,” “financial aspect,” “time spent with the doctor,” and “accessibility and convenience,” was used in this cross-sectional study on 384 patients of two tertiary care facilities in Riyadh, Saudi Arabia, over a 6-month period. The degree to which sociodemographic characteristics and components of patient satisfaction are correlated was assessed using binary and multiple regression analysis. When the *P*-value was < 0.05, the results were considered significant and were presented as adjusted odds ratios (AOR). To ascertain how each PSQ-18 subscale affected other subscales, a Pearson Correlation analysis was conducted. The overall degree of satisfaction with all 18 items was 73.77%. The financial component received a rating of 81% compared to 77% for general satisfaction. Technical quality (75%) was followed by accessibility and convenience (73.5%), communication (73%), and interpersonal elements (72%). At 68%, the time spent in the doctor's domain received the lowest rating. The odds of satisfaction were increased by 3.87 times, 3.45 times, and 3.36 times among those who are employed, qualified by university education, and married compared to unemployed (*P-*value = 0.018), less qualified (*P-*value = 0.015) and singles (*P-*value = 0.026), respectively. The younger age group also made 1.78 times more of a difference in higher satisfaction ratings. The general satisfaction domain showed a positive association with other areas. Participants who were satisfied with the communication and accessibility and convenience domains of healthcare providers were the only ones who were typically satisfied with the domain of doctor time spent. The study's findings could act as a benchmark for Saudi Arabia's healthcare services as well as a starting point for quality assurance procedures.

## Introduction

The idea of the quality of care is crucial to efforts for quality assurance and improvement in healthcare. Although the quality in the healthcare sector has long been recognized as important, patient agendas, quality improvement programs, and quality insurance have recently given it more momentum (1). Although the primary consideration in healthcare is quality of care rather than cost (2), it can be quite challenging for a patient to assess the service provider's technical ability and the immediate effects of numerous therapies (3).

It has been suggested that we can evaluate the effectiveness of healthcare by looking at its organization, procedures, and results (4). While the goals of healthcare efficacy and safety are almost universal, many countries and organizations place a greater or lesser emphasis on the additional goals of patient-centeredness, timeliness, efficiency, and equity. Healthcare metrics, such as process metrics, are created for a variety of audiences that might want to use them for healthcare utilization, purchasing, or performance improvement (5). They must be relevant, scientifically valid, generalizable, and interpretable to serve all these functions (6).

Patient satisfaction is a crucial indicator of the quality of a hospital's care because it provides insight into how well the provider has met the patient's most essential expectations (7) and is a major factor in determining the patients' perspective on their intended behavior (8). Patient satisfaction is associated with significant benefits, including better compliance, a reduction in the use of medical services, fewer malpractice lawsuits, and a better prognosis (7). In the past several years, there have been a plethora of surveys that only focus on patient experience, i.e., aspects of the care experience like waiting times, the quality of basic amenities, and communication with healthcare practitioners, all of which help identify concrete goals for quality improvement. This is due to the lack of a solid conceptual foundation and uniform measuring instrument for customer satisfaction (9).

According to some published literature, defining quality improvement from the perspective of patients provides better value for money by improving the safety, accessibility, equity, and comprehensiveness of treatment, whereas, from the standpoint of providers, quality improvement may be more efficient, offering more effective services to a greater number of customers with a decent degree of satisfaction, with the latter being sufficient for retaining customers (10).

There are numerous published studies that emphasize the importance of patient-related and socioeconomic factors in influencing patient satisfaction with healthcare services. Mummalaneni and Gopalakrishna (11) assert that socio-demographic variables including age, gender, occupation, employment status, education, and income have an impact on patients' satisfaction with healthcare. Additionally, Gordo (12) analyzes data from the German Socio-Economic Panel and discovers that there is a substantial correlation between long-term unemployment and patient satisfaction, whereas there is a minor correlation between short-term unemployment and patient satisfaction depending on gender. Finally, Popescu et al. (13) explore health status in relation to health expenditures and healthcare provisions (hospital beds and physicians per person) and discovers a strong association between reporting a good or bad health status and health expenditures and services. The education level of the participant was identified as a key predictor of patient satisfaction in an Oman study (14).

The Kingdom of Saudi Arabia, which has a land area of 2,250 000 square kilometers and a population of more than 34 million people, is the largest country in the Middle East. Around 25% of its population is made up of foreigners. A sizable portion of the population (60%) is under 25 years old. It is essential to understand patient satisfaction in this heterogeneous group and to evaluate the influence of predictors on patient satisfaction ratings. One study found a significant variation in the level of satisfaction with health services offered by Saudi Arabian primary health centers based on age, gender, presence of chronic health issues, and employment status (15). According to a recent Saudi Arabian report, patients who are employed, highly educated, younger, and male exhibited higher levels of satisfaction with hospital services on the PSQ-18 patient satisfaction measure (16). However, mapping the degree of satisfaction with various clinical and sociodemographic factors and investigating the impact of those factors on patients' satisfaction scale measurements for general satisfaction, technical quality, interpersonal manner, communication, financial aspects, time spent with the doctor, and accessibility and convenience will help in drawing more robust measurements of specific areas that need to be focused for improvement. Therefore, a cross-sectional study was conducted to measure the level of satisfaction of general patients with the health care services provided at tertiary care facilities in Riyadh, Saudi Arabia. Additionally, it aimed to investigate the key areas of the patient satisfaction scale that require immediate intervention and to identify the factors that predict patients' satisfaction.

## Materials and methods

### Study setting

This six-month cross-sectional correlation study was conducted on a systematically random sample of patients using the outpatient departments at two multispecialty hospitals in Riyadh, Saudi Arabia, from October 2020 to March 2021. These health centers provide health care to all citizens and residents who work in the public sector. These tertiary care facilities accept referrals from all other health centers throughout the nation.

### Study participants

The participants from this study were in the age group of 18–60 years receiving treatment in any of the departments of the two selected hospitals in Riyadh, Saudi Arabia. The selected participants were in sufficient mental, physical, and emotional health to provide their permission to take part in the study. Participants with cognitive performance indicating moderate to severe cognitive impairment, who were unable to give consent and were not willing to participate, were excluded from the study.

### Sample size calculation

The required sample size was calculated using the OpenEpi software (http://www.openepi.com/SampleSize/SSPropor.htm) after the level of significance and power were both set at 0.05 and 0.8, with a precision of 95 percent confidence interval. The expected sample size was 384.

### Sampling method

As previously indicated, we used the systematic random sampling method (14). We first compiled a list of the anticipated participants in each facility during the study period, calculated the sampling fraction based on the necessary sample size, and then randomly chose patients from the list of participants using online randomizer software (https://www.randomizer.org/). The next person in line was picked if the chosen participant did not satisfy the requirements for inclusion. Eight participants' responses were incompletely filled out, resulting in only 384 samples being selected for outcome analysis out of a total of 392 samples collected during the study period.

### Study materials

The questionnaire was divided into two sections. The participants' sociodemographic information, including gender, age, education level, employment status, and marital status, were covered in the first section of the questionnaire. A condensed version of the standard patient satisfaction questionnaire (PSQ-18) was included in the second section (17). There are seven distinct evaluation categories that include various facets of satisfaction. Items 2, 4, 6, and 14 evaluate the technical quality of services, while items 3 and 17 assess general satisfaction. While items 1 and 13 determined communications, items 10 and 11 evaluated interpersonal behavior. The financial aspect is screened by items 5 and 7. Items 8, 9, 16, and 18 evaluated accessibility and convenience, whereas items 12 and 15 measured the times spent with the doctor. The responses were tallied using a Likert scale, with a high score of 5 signifying a high degree of satisfaction with the healthcare services. Item stems 1, 2, 3, 5, 6, 8, 11, 15, and 18 were scored in descending order from strong agreement (score 5) to strong disagreement (score 1), whereas, items 4, 7, 9, 10, 12, 13, 14, 16, and 17 were rated in ascending order from strong agreement (score 1) to strong disagreement (score 5).

The survey was translated into Arabic backward and forwards. With the assistance of a team of subject-matter experts, the face and content validity of each questionnaire utilized in the study were examined. To validate the reliability of the Arabic version, internal consistency tests and item-scale correlations were also carried out (18). Pilot research was carried out to determine whether any elements were misunderstood. The internal consistency of the Arabic questionnaire was evaluated, and the reliability of Cronbach's alpha was validated for seven categories, with Cronbach's coefficients ranging from 0.73 to 0.89.

### Data collection

The data collectors approached the selected participants in the study areas and asked them to participate in the study. Participants were made aware of the study's objectives, methodology, potential risks, the voluntary nature of their participation, and the confidentiality of their responses by the data collector. They were informed that they might refuse or stop participating at any time without suffering any consequences. For ease of understanding and comprehension, the participants self-administered the questionnaire in their native Arabic language. A data collector was there, though, in case there was any misunderstanding. The content of the data collection instruments and interviewing techniques were covered in training for data collectors. The research team supervised the process of data collection.

### Ethical approval

The World Medical Association's Declaration of Helsinki (1964–2008) for Ethical Human Research was strictly followed in the conduct of this study. The study protocol was approved by the Research Committee of the College of Pharmacy at AlMaarefa University in Riyadh, Saudi Arabia.

### Statistical analysis

Using descriptive statistics, the patients' sociodemographic features were listed. All sociodemographic data is presented using percentages and frequencies. Based on the ratings given by the participants for each item in the various outcome variables, the satisfaction score was generated. A scale from 1 to 5 was used to calculate the mean of the scores (Likert scale). The satisfaction score was then separated into two categories: dissatisfaction (below the mean) and satisfaction (equal to or above the mean) (16). To determine the correlations between the numerous independent factors and the outcome variables, binary logistic regression analyses were performed (dependent variables). The degree of patient satisfaction as measured by the PSQ-18 score (the dependent/outcome variable) and the independent factors (socio-demographic characteristics such as age, gender, education level, marital status, and employment position) were compared using the adjusted odds ratio. The influence of sociodemographic features on the outcome of each subscale of PSQ-18 was also determined and compared with the non-parametric chi-square test. Further, Pearson correlation analysis was done to determine the impact of each of the subscales of PSQ-18 on the scoring of other subscales. Two-sided *P*-values (P) and 95% confidence intervals were used in all the studies for significance testing. The average scores of items included in each subscale were calculated and presented in **Table 2** as mean ± SD. It was converted into a percentage level of satisfaction using the formula as mean^*^100/5. *P-*values < 0.05 were deemed statistically significant in the final model. All statistical analysis was done using SPSS-IBM 23 statistical package.

## Results

### Sociodemographic characteristics of the participants

A total of 384 individuals were recruited for the study, and of them, 52% were men and 42% were between the ages of 50 and 60 (Table 1). Most of the respondents were employed (59%), married (63%), and well-educated (61%). Participants were recruited from King Saud Medical City (55%) and King Abdulaziz University hospital (45%).

**Table 1:** Sociodemographic features of the participants.

**Variables**	**Number (384)**	**Percentage**
**Gender**		
Male	198	52%
Female	186	48%
**Age**		
18–30	102	27%
30–50	121	31%
50–60	161	42%
**Educational level**		
Higher^*^	234	61%
Secondary	150	39%
**Marital status**		
Married	242	63%
Single	142	37%
**Employment status**		
Employed	228	59%
Student	35	9%
Non-employed	121	32%
**Study center**		
King Saud Medical City	212	55%
King Abdulaziz University Hospital	172	45%

* Graduates and above.

### Patient satisfaction survey using PSQ-18

The participant's rating on each subscale of the PSQ-18 survey at the two study locations in Riyadh is shown in Table 2. The study's participants receive free medical care in government hospitals; therefore, they are quite content with the financial component, which received a high rating (81%) compared to the subscale that measures the amount of time spent with the doctor, which had a lower rating (68%). Technical quality (75%) and accessibility and convenience (73.5%) were regarded as being of excellent quality, and overall satisfaction with medical care (77%) was also high. Additionally, 73 and 72%, respectively, were assigned to the communication and interpersonal components.

**Table 2:** Description of the PSQ-18 in different subscales.

**Serial Number**	PSQ-18 subscale^*^	**Number of items included**	**Item number included**	**Mean ±SD**	**Level of satisfaction (%)**
1.	General satisfaction	2	3,17	3.85 ± 0.46	77
2.	Technical quality	4	2,4,6,14	3.75 ± 0.32	75
3.	Interpersonal aspect	2	10,11	3.6 ± 0.22	72
4.	Communication	2	1,13	3.65 ± 0.11	73
5.	Financial aspect	2	5,7	4.05 ± 0.14	81
6.	Time spent with doctor	2	12,15	3.40 ± 0.23	68
7.	Accessibility and convenience	4	8,9,16,18	3.67 ± 0.36	73.5

* PSQ, Patients satisfaction questionnaire. The average scores of items included in the subscale were presented as mean ± SD; the percentage level of satisfaction was calculated using the formula as mean^*^100/5.

**Figure 1:**
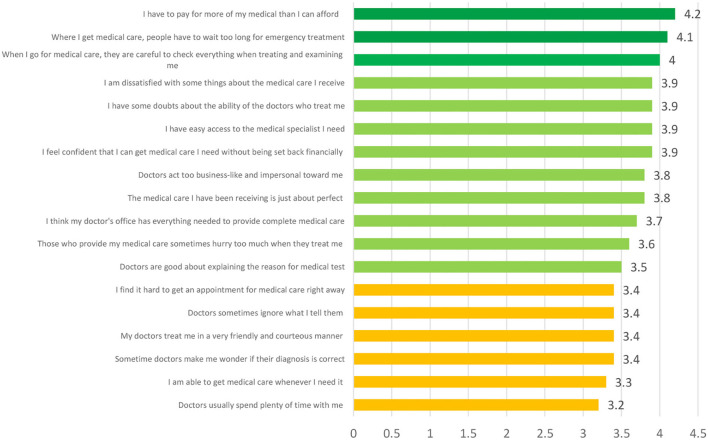
Comparison of scores given by participants of the study on PSQ-18 items.

The participants' ratings on each of the 18 items on the PSQ-18 scales are shown in Figure 1. The participants gave the hospitals' payment policies and medical care a strong recommendation. They feel fairly at ease with the doctors' explanations, the doctor's skills, and the availability of the right medical specialty. Lower scores are given to the patient's satisfaction with the amount of time the doctor spends with them, the quality of their medical care, their trust in the accuracy of their diagnosis, and the time they need to spend scheduling an appointment.

### Comparison of satisfaction of patients with sociodemographic features

The mean of the ratings given by the participants of this study was 3.68 (73.77%) with a standard deviation of 0.28. Therefore, the satisfaction score was divided into two categories: satisfaction (≥3.68), and dissatisfaction (<3.68). Table 3 summarizes the results of the logistic regression study of the factors affecting ratings on the PSQ-18 scale (outcome variable). Participants who were employed or studying had 3.87 and 2.16 times more chances of being satisfied with healthcare services than unemployed individuals, respectively. Surveyors with university degrees were more likely to report being satisfied than those with only secondary education, by a factor of 3.45. Compared to those who were living alone, married status was 3.36 times more favorably associated with patient satisfaction. Additionally, compared to individuals from the older age group, younger participants between the ages of 18 and 30 had a 1.78 times higher likelihood of being satisfied with the healthcare services (50–60 years). In contrast, there is a negative relationship between satisfaction and middle-aged age (30–50 years) as compared to older age groups. In terms of participants' levels of satisfaction with hospital services, there is no significant difference between male and female participants. Finally, there was no significant difference in patient satisfaction between the two research sites.

**Table 3:** Influence of factors on patients' satisfaction using PSQ-18 scale.

**Variables**	**Frequency (%)**		**AOR (95% CI)**	***P-*** **value**
	**Satisfied** **[238 (62)]**	**Dissatisfied** **[146 (38)]**		
**Gender**				
Male	138 (58)	60 (41)	1.61 (0.98, 2.76)	0.043^*^
Female	100 (42)	86 (59)	R	
**Age (years)**				
18–30	72 (30)	30 (21)	1.78 (1.10, 3.39)	0.025^*^
30–50	56 (24)	65 (45)	0.48 (0.34, 0.98)	0.031^*^
50–60	110 (46)	51 (35)	R	
**Educational level**				
Higher	169 (82)	65 (61)	3.45 (1.76, 4.68)	0.015^*^
Secondary	69 (18)	81 (39)	R	
**Marital status**				
Married	167 (70)	75 (51)	3.36 (1.65, 5.21)	0.026^*^
Single	71 (30)	71 (49)	R	
**Employment status**				
Employed	163 (68)	65 (45)	3.87 (1.92, 5.12)	0.018^*^
Student	27 (11)	8 (5)	2.16 (1.12, 3.5)	0.021^*^
Non-employed	48 (20)	73 (50)	R	
**Study center**				
King Saud Medical City	135 (57)	77 (53)	1.09 (0.87, 1.21)	0.68
King Abdulaziz University Hospital	103 (43)	69 (47)	R	

R, showing the reference used.

* P < 0.05 in the multivariate analysis and statistically significant.

CI, Confidence interval; AOR, Adjusted odds ratio; PSQ, Patient satisfaction questionnaire.

### Comparison of sociodemographic features with subscales of PSQ-18

Table 4 demonstrates that individuals with higher education scored significantly (*P* < 0.05) higher on the PSQ-18 subscales of general satisfaction, technical quality, and financial factors than participants with lower education. Furthermore, married people scored significantly (*P* < 0.05) better score on the PSQ-18 general satisfaction, technical quality, and financial component subscales than single people. Participants who were employed or enrolled in education also scored significantly (*P* < 0.05) higher score in the general satisfaction, technical quality, and financial aspect categories than those who were unemployed.

**Table 4:** Comparison of PSQ-18 subscales with sociodemographic factors.

**Serial number**	**General satisfaction**	**Technical quality**	**Interpersonal aspect**	**Communication**	**Financial aspect**	**Time spent with doctor**	**Accessibility and convenience**
**Gender**							
Male	3.94 ± 0.6	3.87 ± 0.5	3.7 ± 0.8	3.80 ± 0.1	4.3 ± 0.4	3.6 ± 0.4	3.8 ± 1.1
Female	3.76 ± 0.4	3.63 ± 0.2	3.6 ± 0.3	3.50 ± 0.2	3.8 ± 0.4	3.2 ± 0.5	3.54 ± 1.2
**Age (years)**							
18–30	3.84 ± 0.3	3.95 ± 0.1	3.71 ± 0.3	3.74 ± 0.9	4.21 ± 0.5	3.65 ± 0.6	3.80 ± 1.3
30–50	3.60 ± 0.2	3.54 ± 0.2	3.44 ± 0.2	3.46 ± 0.8	3.76 ± 0.7	2.98 ± 0.7	3.42 ± 1.2
50–60	3.85 ± 0.1	3.76 ± 0.3	3.65 ± 0.1	3.75 ± 0.8	4.18 ± 0.8	3.57 ± 0.7	3.79 ± 1.1
**Educational level**							
Higher	4.12 ± 0.1^*^	4.22 ± 0.3^*^	3.80 ± 1.0	3.82 ± 0.7	4.24 ± 0.8^*^	3.76 ± 0.6	3.92 ± 1.1
Secondary	3.58 ± 0.2	3.28 ± 1.2	3.40 ± 1.1	3.48 ± 0.6	3.86 ± 0.9	3.04 ± 1.1	3.42 ± 0.8
**Marital status**							
Married	4.10 ± 0.4^*^	4.05 ± 1.1^*^	3.90 ± 1.1	3.91 ± 0.5	4.35 ± 0.8^*^	3.81 ± 0.5	3.89 ± 0.8
Single	3.60 ± 0.6	3.45 ± 0.2	3.30 ± 0.2	3.39 ± 1.2	3.75 ± 1.1	2.9 ± 0.9	3.45 ± 0.8
**Employment status**							
Employed	4.18 ± 0.4^*^	3.95 ± 0.3^*^	3.81 ± 0.7	3.95 ± 1.1	4.12 ± 0.3^*^	3.74 ± 0.3	3.85 ± 0.7
Student	4.1 ± 0.3^*^	4.02 ± 0.4^*^	3.74 ± 0.6	3.88 ± 1.1	4.23 ± 0.7^*^	3.71 ± 0.6	3.79 ± 0.6
Unemployed	3.27 ± 0.3	3.28 ± 0.3	3.25 ± 0.4	3.12 ± 0.4	3.80 ± 0.8	2.75 ± 0.5	3.37 ± 0.5
**Study location**							
King Saud Medical City	3.90 ± 0.1	3.70 ± 0.6	3.70 ± 0.8	3.60 ± 0.6	4.11 ± 0.3	3.45 ± 0.4	3.71 ± 0.3
King Abdulaziz University Hospital	3.80 ± 0.2	3.80 ± 1.1	3.50 ± 0.9	3.72 ± 0.5	3.99 ± 0.1	3.35 ± 0.3	3.63 ± 0.9

* P-value < 0.05 using chi-square test.

### Correlation between subscales of the PSQ-18 scale

Pearson correlation analysis was done to determine the impact of each subscale's scores on the results of the other subscales. Table 5 illustrates that participants are more likely to perform well on the technical quality, communication, financial aspect, and accessibility and convenience subscales if they score well on the general satisfaction subscale. A participant's likelihood of scoring well on the interpersonal aspects' subscale is higher when they receive higher technical quality scores. Furthermore, a positive association was found between the interpersonal and financial aspects, as well as the time spent with the doctor subscales. Additionally, the accessibility and convenience subscales and time spent with the doctor were all positively correlated with the communication subscale. The interpersonal and communication subscales were also linked to the financial element subscale. The subscales of interpersonal factors, communication, financial aspects, and accessibility and convenience were all well-performed by participants who did well on the subscale “time spent with the doctor.” Finally, those who had better accessibility and convenience were able to spend more time with the doctor.

**Table 5:** Correlation between subscales of PSQ-18.

**Variables**	**Correlation coefficient (r)**						
	**General satisfaction**	**Technical quality**	**Interpersonal aspect**	**Communication**	**Financial aspect**	**Time spent with doctor**	**Accessibility and convenience**
General satisfaction	—	0.123	0.144^*^	0.032	0.031	0.034	0.112
Technical quality	0.145^*^	—	0.061	0.213^*^	0.024	0.056	0.012
Interpersonal aspect	0.123	0.232^*^	—	0.231	0.453^*^	0.231^*^	0.034
Communication	0.151^*^	0.125	0.092	—	0.221^*^	0.332^*^	0.021
Financial aspect	0.155^*^	0.089	0.256^*^	0.012	—	0.257^*^	0.022
Time spent with doctor	0.067	0.091	0.266^*^	0.543^*^	0.045	—	0.233^*^
Accessibility and convenience	0.162^*^	0.081	0.191	0.234^*^	0.056	0.231^*^	—

* P-value < 0.05.

## Discussion

Saudi Arabia is undergoing a massive transformation in healthcare delivery with the implementation of the health sector transformation program as part of the Saudi Vision 2030. The program intends to redesign the Kingdom's health system so that it is comprehensive, efficient, and integrated based on the well-being of each person and the health of the community. By applying and adhering to the finest evidence-based worldwide practices that secure and make use of the greatest services and care for society, this system is focused on the satisfaction of the beneficiaries. The two major objectives of this program are facilitating access to healthcare services and improving the quality and efficiency of those services (19). Our research sought to ascertain the degree of satisfaction with the medical care provided at reputable tertiary care facilities in Saudi Arabia's capital, Riyadh. We hope and believe that the results of this study will provide policymakers and strategic planners with first-hand information about the key areas that need to be prioritized to offer the best healthcare possible to society.

The overall level of satisfaction as determined by the PSQ-18 was found to be 73.77%, which is marginally higher than the results of the earlier study (16), which was conducted in Riyadh patients with psychological disorders. In terms of the factors associated with patients' satisfaction with healthcare services, we found that highly educated, employed, participants between the ages of 18 and 30 years, and married individuals had higher levels of satisfaction than less educated, single, participants over the age of 50 years, and unmarried/single individuals, respectively.

In keeping with an earlier study (20), we found that participants in our study samples with a university education were satisfied with the health services given in the medical settings. This may be because participants with higher education levels are more likely to be aware of the hospital's offerings than those with lower education levels. Our report corroborates with Karaca and Durna, who observed that a patient's degree of education is related to their satisfaction with care; in their study, people with a college or university education were found to be more satisfied with care (21). According to published literature (22), higher education aids in the development of greater responsiveness to the care and services provided by the healthcare system, which ultimately may result in improved satisfaction ratings.

This study's high degree of satisfaction among employed people was an additional interesting finding. We found that participants who were employed reported higher levels of satisfaction with hospital-provided healthcare as compared to research participants who were not employed. Our findings are in line with those of Aloh et al. (10), who reported a substantial correlation between patients' overall satisfaction with the quality of care offered at tertiary care facilities in Southeast Nigeria and their employment status. The findings of Myers et al. (23) and Bernheim et al. (24) that physicians as a group perceive and treat unemployed patients differently and at a lower level could not be corroborated by us. There may not be a direct correlation between the treatment provided to patients by doctors and their employment status because the ministry of health covered all expenses for care at our study center. Therefore, we hypothesize that the low level of patient satisfaction among unemployed patients may be related to their perception of the discrepancy theory, according to which patients with low social status may believe they have been denied the services and care that are typically provided to those in higher social classes.

Another complicated relationship we correlated in this study was between the age of the participants and their satisfaction rating on health services. Patient satisfaction percentages may vary by age group; therefore, this might be the case (25). Participants under the age of 30 years expressed greater satisfaction with the hospital's services than participants over the age of 50 years. A study found a direct relationship between age and patient satisfaction up to about 65 to 80 years and subsequently declines (26). In our study, we discovered that patient satisfaction was a high when they were <30 years old, but between 30 and 50 years old, it started to fall. The low score suggests high expectations that cannot be readily realized from various hospital units, and it is conceivable that patients in this age group visit health centers more frequently for the medical treatment of their family members, including their children and spouse. However, as patients age and become more accustomed to receiving medical treatment, their expectations may reduce along with the number of visits they require for their family's needs. This presumably leads to a higher level of satisfaction (26). Due to their age-related morbidities, visits to the medical facility grow as people get older (more than 60–65 years), and there is a likelihood that their expectations will also increase. We are unable to show a change in satisfaction with advancing years because the study's participants were only up to the age of 60 years.

Although we found a difference between men and women on the level of satisfaction, it was not significant. It is possible for men and women to use services in different ways, to perceive things differently, and to have different needs and expectations from the healthcare services that are offered. The satisfaction levels of men and women may differ, which may reflect these inequities. This investigation's findings are consistent with the previous report (27). Our findings are contrary to earlier reports by Alhusban and Abualrub (28) and Zarzycka et al. (29), where they found a higher rate of satisfaction of female patients with the healthcare services, however, most of the items used in those surveys were meant to elucidate the nursing care satisfaction and generally female patients are more satisfied with nursing care than other health care services as we reported earlier in Saudi Arabia (16). In line with our findings, other reports (30, 31) did not agree that there is any influence of gender on patients' satisfaction.

In congruent with previously published literature (32), we found a beneficial influence of marriage on their level of satisfaction with health care services. There is a growing body of literature on the topic of marriage protection. This idea makes use of a solid social network to raise customer satisfaction with the services provided. In contrast, a study found a negative correlation between marriage and satisfaction with healthcare services (33); perhaps because a large portion of the participants in this study had low levels of education and was either unemployed or retired, it is possible that other factors in addition to marital status may have a concurrent impact on the level of satisfaction.

The current study found that the PSQ-18's financial element subscale received the strongest patient support, while the time spent with the doctor had the weakest support. It is hardly surprising that the participants expressed strong support for finance. All study participants received free medical treatment, and because Saudi Arabia is a high-income nation, most pharmaceuticals that are available in developed nations are also easily accessible here. Patients are enraged by the length of time they must wait for emergency care. They have expressed dissatisfaction with the diagnoses provided by doctors. They also find it frustrating that they can't get appointments with doctors and that the doctors don't seem to care about their problems. Prior research has demonstrated that patient satisfaction is significantly predicted by information intake, listening, empathy toward the patient, emotional support, friendliness, explanation of medical therapy, and respect for the patient (34). Additionally, the use of medical words by doctors without defining them negatively impacts patient satisfaction (35). In terms of the right to respect, investigations conducted in developing countries have revealed that patients are willing to endure disrespect from doctors. It can be an example of the “paternalistic approach,” a traditional viewpoint that holds that patients are beneath doctors (36). An earlier study (37) conducted in Saudi Arabia identified a lack of time and communication from the doctors as a significant factor in their lower level of satisfaction with healthcare services. While the majority of PSQ18 survey items had medium-level ratings, the financial and general satisfaction domains did better when contrasted with other domains. Furthermore, with satisfaction rates of 61.2 and 60%, respectively, in Ethiopia (27) and Austria (36), our study is comparable to those studies. In general, most patients were disappointed with the amount of time they spent with the doctor. They often schedule a visit far in advance and want to have a long talk with the doctor, but given the doctor's busy schedule, this is not practical. Additionally, correlation analysis demonstrated that there is a substantial correlation between several of the PSQ-18 subscales' domains. Participants who evaluated positively on the general satisfaction domain rated highly on most of the other domains, whereas those who rated highly on the technical quality domain also rated better on the interpersonal domain. Participants who were satisfied with the healthcare providers' communication and the accessibility and convenience domains were the only ones who were usually satisfied with the domain of doctor time spent.

Finally, these results may have been influenced by several confounding variables. Additionally, since this was a cross-sectional study done at a certain time, it's likely that some of the respondents were just beginning their treatment while others had a long history of relationship with the hospital and were therefore confident in the quality of the services. As a result, the data do not permit inference as to whether the extent of care received at the hospital influences satisfaction rating. Prior research, however, indicated that the patient's underlying condition was more likely to have an impact on satisfaction than the length of hospital treatment (38).

## Conclusion

The method of evaluating patient satisfaction promotes a quality ethos by informing patients that physicians are held accountable, as well as rewarding physicians by providing them with the knowledge that patients are satisfied with the level of treatment they receive. Patients seeking consultations in tertiary care facilities appear low in satisfaction with the amount of time spent with the doctor and the quality of the consultation. Additionally, they had issues with service accessibility and convenience. Age, employment status, marital status, and level of education all had a beneficial impact on patients' satisfaction. Examining additional drivers of care quality, or elements from the provider side that can affect quality, is necessary. In addition, an in-depth review of inpatient satisfaction with specific hospital services is required to know the overall status of satisfaction.

## Data availability statement

The original contributions presented in the study are included in the article/supplementary material, further inquiries can be directed to the corresponding author.

## Ethics statement

The study protocol was approved by the Research Committee of the College of Pharmacy at AlMaarefa University in Riyadh, Saudi Arabia. Written informed consent for participation was not required for this study in accordance with the national legislation and the institutional requirements.

## Author contributions

Under the supervision of SAs, AK, RA, WA, MAlm, AA, and FAlo carried out the research methodology. NF, FAls, and MAls were responsible for the formal analysis of the work while MAlh and SAls participated in writing the original draft of the manuscript. MAlm administered the project. SAs was instrumental in reviewing and editing of the manuscript. All authors contributed to the article and approved the submitted version.
